# Preparedness of South Asian countries regarding Langya virus emergence: A view on the current situation

**DOI:** 10.1002/hcs2.42

**Published:** 2023-04-18

**Authors:** Al Kamal Muhammad Shafiul Kadir, Tungki Pratama Umar, Abdullah Al Rabbi, Md. Suza Chowdhury, Mohammad Ullah Shemanto

**Affiliations:** ^1^ Biomedical Research Division, Institute of Biological Sciences (IBSc) University of Rajshahi Rajshahi Northern Region Bangladesh; ^2^ Medical Profession Program, Faculty of Medicine Sriwijaya University Palembang South Sumatera Indonesia; ^3^ Health Education Division Central Medical Assistant Training School Rangpur North Bengal Bangladesh; ^4^ Health Education Division Smart Living Nursing College Rangpur North Bengal Bangladesh; ^5^ Healthcare Services Division Ahsania Mission Cancer and General Hospital (AMCGH) Dhaka Central Region Bangladesh

**Keywords:** healthcare, henipavirus, public health, South Asia

**Abbreviation**
LayV: Langya Henipavirus

## 1 INTRODUCTION

Langya Henipavirus (LayV) is a zoonotic virus that has recently re‐emerged in Eastern China. There is limited data available on the global prevalence, mortality, and morbidity of LayV. The majority of cases have been reported in two provinces in China (Shandong and Henan). Between 2018 and 2022, 35 individuals were diagnosed with the LayV infection in these regions. This novel ribonucleic acid virus attacks animals (including goats, dogs, and shrews) and can potentially spread to humans. The Henipavirus genus is a Paramyxoviridae family member that can be spread through animal‐human contact with no established human‐to‐human transmission yet [1].

There are several disease spectra associated with LayV, including fever, fatigue, nausea, vomiting, cough, and headache. Furthermore, 34% of patients had elevated liver function tests, and 8% had impaired kidney function [2]. Henipaviruses are biosafety level 4 microbes that have the potential to sicken both humans and animals severely [1]. One of its species, the Nipah virus, can cause serious infection in the presence of acute respiratory infection and fatal encephalitis, with a case fatality rate ranging from 40% to 75% [3].

## 2 POTENTIAL CHALLENGES OF HENIPAVIRUS MANAGEMENT TO THE GLOBAL HEALTHCARE SYSTEM

Currently, Henipavirus is not treatable with medications or protected against by vaccinations; thus, the treatment for human infection is mainly supportive, like other viruses including during the current COVID‐19 pandemic. The need of raising public understanding of many elements of emerging viruses cannot be overstated, for scientists to continue to monitor the emergence of zoonotic diseases, and to practice rigorous personal hygiene due to a potentially lethal consequence of the virus spread [4].

With any emerging virus, it is important to have full genome detection, determine the mode and risk of transmission, and study possible therapeutics and preventive therapies for regional preparedness. LayV is a newly discovered member of the Henipavirus family, which also includes Nipah and Hendra viruses. It is important to consider the potential challenges that Henipaviruses, including LayV, may pose to the global healthcare system [1]. Some potential challenges include limited diagnostic and surveillance tools, high fatality rates, zoonotic transmission, potential for person‐to‐person transmission, and lack of awareness and preparedness among healthcare providers and public health officials.

## 3 SOUTH ASIA REGION AND THE RISK OF CONTRACTING LANGYA VIRUS

The incidence of zoonotic viral infections in South Asia is multifaceted and versatile: diseases that are old, emergent, re‐emerging, and those that have just been found. They all coexist and interplay in unique ways that we are still learning more about. South Asia is frequently believed to be a crucial hotspot for zoonotic spread to humans. Human behavior, large human and animal demographic density, intense animal‐human interaction, multiple live animal marketplaces, urbanization, deforestation, and fragile ecosystems may often raise the likelihood of zoonotic disease development and transmission. This is proved during the COVID‐19 pandemic, which has identified the weakness of South Asia's infection control system, such as by becoming the epicentrum of Delta variant in the middle of 2021 [5].

Furthermore, although the COVID‐19 pandemic has not yet finished, a multicountry epidemic of monkeypox, a zoonotic viral disease, is already spreading globally, including in South Asia [6]. In addition to monkeypox, South Asia is also prone to other zoonotic infections such as Nipah virus, Crimean‐Congo hemorrhagic fever, and chikungunya. For example, in May 2018, a Nipah virus outbreak in India had a high mortality rate, with 88.8% of those affected succumbing to the disease [7]. While there is currently no evidence to suggest that the LayV poses a pandemic threat, it is crucial for researchers to continue monitoring and investigating people and animals to prevent its spread [4]. Therefore, tailored management strategies that are specific to the disease must be put in place.

Although the newly emerging LayV has yet to affect South Asia, it may happen in the future due to cross‐border social and economic ties with China. The risks imposed by zoonotic diseases are extensive; however, they are particularly evident in low and middle‐income countries, where various socioeconomic, environmental, and other situational factors frequently intersect to raise outbreaks of infectious diseases risks and vulnerabilities. These causes include unstable and under‐resourced healthcare systems, fast‐growing populations, rapid industrialization, varying literacy, economic vulnerability, bush meat intake, and human encroachment on animal habitats due to agriculture and other land use changes. A recent study discovered that the danger of zoonotic disease spread is enhanced in tropical places, particularly the Indian subcontinent, that are undergoing agricultural land use changes and have significant animal biodiversity [8].

The current knowledge of the current LayV epidemiology and transmission risk still needs to be increased due to the unfamiliarity of the observation and the dearth of cases reported thus far. Combating new developing zoonotic illnesses in the South Asian region will require a focus on immunization and preventive disease initiatives and a comprehensive review of these infections through research. As a result, it is important to form cross‐border collaborations with global organizations to combat the LayV epidemic at its root [9].

## 4 STEPS OF SOUTH ASIA REGION PREPARATION AGAINST POTENTIAL SPREADING OF LANGYA VIRUS

To prepare for the potential spread of the LayV in South Asia, it is crucial to have a comprehensive understanding of transmission pathways, pathophysiology, reservoir hosts, vulnerable hosts, viral surface survival and stability, rapid diagnostic competencies, and efficient therapies. Therefore, more studies and monitoring of LayV are needed, particularly given the current gaps in information and the potential public health implications of zoonotic infections [3]. Some practical recommendations for the public, governments, and regional integration efforts to prepare for and prevent the spread of LayV may include:

- **Public awareness and education:** Governments should provide accurate and timely information about the virus and its transmission to the public [10]. Public health officials should work closely with communities to raise awareness about the importance of hand hygiene, avoid contact with sick animals, and visit medical care promptly if there is a suspected disease symptoms.
- **Strengthening surveillance and monitoring:** Governments in the South Asia region should strengthen their surveillance and monitoring systems for Henipaviruses [11]. This includes monitoring wildlife populations, identifying and reporting potential cases of Henipavirus infection in animals, and establishing laboratory capacity for virus testing.
- **Enhancing capacity for outbreak response:** Governments should enhance their capacity for outbreak response, including developing and implementing emergency response plans, training healthcare workers and emergency responders, and establishing effective communication systems to ensure rapid response [12].
- **Regional collaboration and integration:** Regional collaboration and integration can play a vital role in preventing and controlling the spread of LayV [13]. Governments in the South Asia region should work together to share information and resources, harmonize surveillance and response efforts, and establish regional centers of excellence for research and capacity building.
- **Research and development:** Governments and international organizations should prioritize research and development for Henipaviruses, including LayV. This includes developing effective diagnostic tests, vaccines, and therapeutics, and investing in basic research to better understand the virus and its transmission [14].

Preventing and combating the outbreak of LayV will require a multifaceted approach involving public awareness and education, strengthening surveillance and monitoring, enhancing capacity for outbreak response, regional collaboration and integration, and research and development [15]. By working together, governments, public health officials, and communities can help to prevent the spread of this and other emerging infectious diseases in the region.

## 5 CONCLUSION

The discovery of LayV in Eastern China necessitates ongoing local epidemiology and surveillance in other hosts besides people. The fact that it has been found in a variety of animal species and people, in addition to the few cases of infection that have been reported so far though, necessitates additional research into the disease's immediate and long‐term spread. Finally, the newly identified LayV infections underscore the vital value of zoonotic surveillance on a worldwide scale to identify the entry and spread of novel zoonotic agents in humans.

## AUTHOR CONTRIBUTIONS

**Al Kamal Muhammad Shafiul Kadir**: conceptualization (lead); data curation (equal); validation (equal); visualization (equal); writing—original draft (equal). **Tungki Pratama Umar**: data curation (equal); formal analysis (equal); project administration (equal); supervision (equal); validation (equal); writing—review & editing (equal). **Abdullah Al Rabbi**: data curation (equal); investigation (equal); project administration (equal); resources (equal). **Md Suza Chowdhury**: data curation (equal); investigation (equal); methodology (equal); project administration (equal); software (equal). **Mohammad Ullah Shemanto**: data curation (equal); project administration (equal); software (equal); writing—original draft (equal).

## CONFLICT OF INTEREST STATEMENT

The authors declare no conflicts of interest.

## ETHICS STATEMENT

Not applicable.

## INFORMED CONSENT

Not applicable.

## Data Availability

Not applicable.
